# Exogenously Used 24-Epibrassinolide Promotes Drought Tolerance in Maize Hybrids by Improving Plant and Water Productivity in an Arid Environment

**DOI:** 10.3390/plants10020354

**Published:** 2021-02-12

**Authors:** El-Sayed M. Desoky, Elsayed Mansour, Mohamed M. A. Ali, Mohamed A. T. Yasin, Mohamed I. E. Abdul-Hamid, Mostafa M. Rady, Esmat F. Ali

**Affiliations:** 1 Botany Department, Faculty of Agriculture, Zagazig University, Zagazig 44519, Egypt; Sayed1981@zu.edu.eg; 2 Department of Crop Science, Faculty of Agriculture, Zagazig University, Zagazig 44519, Egypt; sayed_mansour@zu.edu.eg (E.M.); mohammed_ali@zu.edu.eg (M.M.A.A.); mayasein@zu.edu.eg (M.A.T.Y.); mielsayed@zu.edu.eg (M.I.E.A.-H.); 3 Botany Department, Faculty of Agriculture, Fayoum University, Fayoum 63514, Egypt; 4 Department of Biology, College of Science, Taif University, P.O. Box 11099, Taif 21944, Saudi Arabia; a.esmat@tu.edu.sa

**Keywords:** irrigation water deficit, brassinosteroids, physiological parameters, water output of yields, yield-contributing traits

## Abstract

The influence of 24-epibrassinolide (EBR_24_), applied to leaves at a concentration of 5 μM, on plant physio-biochemistry and its reflection on crop water productivity (CWP) and other agronomic traits of six maize hybrids was field-evaluated under semi-arid conditions. Two levels of irrigation water deficiency (IWD) (moderate and severe droughts; 6000 and 3000 m^3^ water ha^−1^, respectively) were applied versus a control (well-watering; 9000 m^3^ water ha^−1^). IWD reduced the relative water content, membrane stability index, photosynthetic efficiency, stomatal conductance, and rates of transpiration and net photosynthesis. Conversely, antioxidant enzyme activities and osmolyte contents were significantly increased as a result of the increased malondialdehyde content and electrolyte leakage compared to the control. These negative influences of IWD led to a reduction in CWP and grain yield-related traits. However, EBR_24_ detoxified the IWD stress effects and enhanced all the above-mentioned parameters. The evaluated hybrids varied in drought tolerance; Giza-168 was the best under moderate drought, while Fine-276 was the best under severe drought. Under IWD, certain physiological traits exhibited a highly positive association with yield and yield-contributing traits or CWP. Thus, exogenously using EBR_24_ for these hybrids could be an effective approach to improve plant and water productivity under reduced available water in semi-arid environments.

## 1. Introduction

Irrigation water deficiency (IWD) is a substantial abiotic stress factor negatively affecting the growth and productivity of different crops. It is linked to the reduction of arable land and food production [1], as well as livestock raising around the world. It causes changes in the indices of plant morphology, physio-biochemistry, including the antioxidant defense system, and molecular biology of plants [2,3,4,5,6,7]. Located in dry regions, more than 50% of the world’s agricultural sector lands face climate changes that increase the frequency of extreme water shortage conditions [8,9,10]. Crop plants attempt to tolerate IWD by avoiding dehydration by retaining a higher amount of water through many plant strategies (e.g., reduced leaf area, stomatal closure, and older leaf senescence), so it rarely corresponds to high yield, and/or by tolerating dehydration by functioning under the IWD event [11,12]. An innate inconsistency between the accumulation of biomass and avoidance of stress has been demonstrated, because the low transpiration rates affect the acquisition of photo-assimilate that depends on the stomatal aperture and leaf area [12,13]. Additionally, increased output and large sinks of plants—especially in cereals, at least—add a burden to the shoot system regarding the status of plant water and maintenance of cell turgor under the adverse conditions of IWD [11]. As a consequence, while the improved plant’s production potential may lead to a preferred performance with stress, it also monitors the increased demand for useful resources, including water. Thus, under IWD, a plant’s high water uptake ability may occasionally increase the frequency of the stress experience. Appropriately, the higher return potential should correlate with a boosted tolerance to stress [12]. IWD causes pigment degradation, thus reducing the chlorophyll content as a result of excessive reactive oxygen species production [14,15]. It reduces carbon assimilation and the photosynthetic electron transport chain, thereby increasing photoinhibition [16,17,18]. It also increases the NADPH and NADPH/NADP+ and decreases the photosynthetic efficiency and NADPH dissipation in chloroplasts, which also reduces the photosynthetic electron transport chain [19,20].

As a remarkable cereal crop, maize (*Zea mays* L.) ranks first in terms of total production and second concerning the area planted, after wheat, worldwide [21]. It is a sensitive crop to drought, and its production is affected destructively by IWD [22]. With the expected rise of IWD in the coming time period due to climate change, this stress will primarily threaten the stability of the management and production of maize crops [9,10,23,24]. Therefore, it is very important to find approaches to elevate IWD tolerance in maize to enhance plant growth and production [25,26]. The most efficient approach to solve the problem is developing drought-tolerant maize hybrids, which needs a profound comprehension of the mechanisms in drought stress responses in maize. However, developing drought-tolerant hybrids require considerable effort, a long time, and high economical investments [26,27]. Therefore, some approaches can be helpful in this regard [6,26,28,29], including the use of plant hormones to enhance plant performance and output under IWD conditions [30,31,32,33,34].

As perceived at the cell surface and maybe biosynthesized in the endoplasmic reticulum, brassinosteroids (BRs) are a class of plant hormones (polyhydric steroids) with a considerable growth-promoting effect. They exert their effect as multidimensional regulators of a plant response to various stresses, as well as the growth and development of different crops [35]. As one of the most important BRs and a byproduct of brassinolide biosynthesis, 24-epibrassinolide (EBR_24_) is a beneficial active molecule in plants. It has been suggested that EBR_24_ is biosynthesized either through an independent or a dependent pathway, as both are compostanol-based [36]. Although its accurate mechanisms are still mysterious [36], it has the potential to improve growth and development and stimulate the different metabolic processes of plants, including CO_2_ fixation, photosynthesis, and protein and nucleic acid biosynthesis [37], through activating many enzymes related to photosynthesis and the components of the antioxidant defense system (e.g., free proline, CAT, POD, and SOD) [35,38] to help alleviate stress conditions, especially drought adversities in different plants [36,39,40]. Several works have been reported stating that the exogenous use of EBR_24_ can increase drought tolerance in plants by enhancing the morphological and physiological responses, including photosynthetic pigments biosynthesis, chlorophyll fluorescence, photosynthetic and photochemical activity, gas exchange indices (i.e., photosynthesis and stomatal conductance and transpiration rates), and the plant water status, which reflects positively in plant productivity [33,34,38,41]. Although they are widely distributed throughout plants, BRs are not transported over long distances among different tissues. However, BRs may have an indirect role in long-distance signals through their influence on other plant hormones. Besides, it has been reported that BRs regulate plant stress tolerance through modulation of the ROS signal, which is also involved in the systemic stress response [42].

Numerous studies have indicated that EBR_24_ promoted the growth of drought-stressed plants. However, information on the mechanisms of growth and yield enhancement induced by EBR_24_ in maize hybrids is not available. Therefore, the current work aimed at determining the fruitful role of EBR_24_ used exogenously in boosting drought tolerance in six commercial maize hybrids (i.e., Giza-162, Giza-166, Giza-167, Giza-168, Giza-176, and Fine-276). This aim can be achieved under semi-arid conditions by exploring improvements in growth, yield, crop water productivity (CWP), physiological traits, and the plant antioxidant system with the application of EBR_24_ to these contrasting hybrids in drought tolerance.

## 2. Materials and Methods

### 2.1. Experimental Site and Agricultural Practices

Using the area designated for maize production in the Experimental Farm of the College of Agriculture, University of Zagazig (30°36′ N, 32°16′ E), Egypt, two summer field trials were implemented in 2018 and 2019. This region is semi-arid, and its climate is characterized by high temperatures and a lack of rainfall during the summer seasons [43]. During 2018 and 2019, Table 1 displays the average monthly temperature (minimum and maximum), growing degree days, and total precipitation, plus the long-term averages of 35 years. Before sowing in both seasons, some chemical and physical properties were analyzed using soil samples collected from 0–30 and 30–60-cm depths. These analyses indicated that the soil was sandy throughout the profile (91.4% sand, 2.7% silt, and 5.9% clay), with 1.63 g cm^−3^ as the average bulk density. Calcium carbonate, organic matter, pH, and electrical conductivity were 5.8 g kg^−1^, 6.1 g kg^−1^, 7.9, and 0.7 dS m^−1^, respectively. Soluble cations and anions were 1.8, 0.25, 1.2, 1.9, 2.2, and 1.6 mmolc L^−1^ for Ca^2+^, Mg^2+^, Na^+^, K^+^, HCO_3_^−^, and Cl^−^, respectively. Additionally, the available nutrients were 23.4-mg N, 6.1-mg P, and 72.3-mg K kg^−1^ of the soil. According to the optimal period of maize cultivation in the region, the seed sowing was performed on the first of May in both seasons. As recommended, the standard agronomic practices, including drip irrigation and control of pests and diseases, were applied for growing maize in the region. Before sowing, 33-kg P unit per ha were added as Ca (H_2_PO_4_)_2_ (15.5% P_2_O_5_), and 95-kg K unit per ha were added after thinning as K_2_SO_4_ (48% K_2_O). Additionally, 300-kg N unit ((NH_4_)_2_SO_4_; 21% N) per ha was applied in five splits at 7-day intervals after sowing.

### 2.2. Plant Material and Irrigation Regimes

For this study, six single-cross hybrids (*Zea mays* L.) were used: Giza-162, Giza-166, Giza-167, Giza-168, Giza-176, and Fine-276. These hybrids are the most common commercial yellow single-cross hybrids that appear in the Egyptian recommended list and have different genetic backgrounds. With three replications for each treatment, a spilt-split plot design was applied to the experiments. The irrigation regimes were specified to the main plots, while foliar treatments and the six evaluated hybrids were randomly distributed into subplots and sub-subplots, respectively. Each plot contained five rows, each 4-m-long and with 0.70 m between the rows, of which plants were 0.25 m apart. This distance design collected a plant density of 57,143 plants ha^−1^. In rows, 3 seeds were sown per hill, and at full emergence (20 days after sowing; DAS), only one seedling (the strongest) was kept. The optimum amount of irrigation water of field crops based on their water requirements, climatic variables, and soil type in different regions in Egypt is identified annually by the Department of Water Requirement and Field Irrigation, Center of Agricultural Research that belongs to the Egyptian Ministry of Agriculture and Land Reclamation. The recommended amount of water for maize under sandy soil in the Elkhatara region is 9000 m^3^ ha^−1^. Therefore, maize hybrids were evaluated under three water irrigation regimes; well-watered (9000 m^3^ ha^−1^; 850, 3500, and 4650 m^3^ for sowing to emergence (1 May–11 May), plant developing to tasseling (12 May–25 June), and tasseling to maturity (26 June–19 August), respectively); moderate drought (6000 m^3^ ha^−1^; 850, 2200, and 2950 m^3^ for sowing to emergence (1 May–11 May), plant developing to tasseling (12 May–25 June), and tasseling to maturity (26 June–19 August), respectively); and severe drought (3000 m^3^ ha^−1^; 850, 900, and 1250 m^3^ for sowing to emergence (1 May–11 May), plant developing to tasseling (12 May–25 June), and tasseling to maturity (26 June–19 August), respectively). From the establishment of seedlings, drought conditions were started up to maturity. The irrigation frequency was once every two days. A drip irrigation system was applied for the experiments. The drip laterals and emitters were spaced at 0.7 and 0.3 m, respectively. The operating pressure and emitter flow rate were maintained at 1 bar and 4 L h^−1^, respectively, by specifying a valve and pressure gauge to each irrigation sector. For each irrigation regime, the targeted amount of water was assessed by using a flow meter. Two weeks before harvesting, irrigation was terminated in both seasons.

### 2.3. Foliar Application

Ethyl alcohol (1 mL) was used to dissolve the 24-epibrassinolide hormone (EBR_24_) to prepare a stock solution, and double-distilled water was used to maintain the final volume. The EBR_24_ was used in three foliar sprays (at 20, 35, and 55 DAS) using a 5-μM rate. The 5-µM concentration was selected according to a preliminary study, where 1, 5, 10, 20, and 40 µM of EBR_24_ were created, and the 5-µM concentration induced the best response in the plants (data not shown). Using a 20-L dorsal sprayer (Beijing, China), the EBR_24_ solution provided with a surfactant (0.001% Tween 20; to promote solution retention on leaves) was sprayed early in the morning onto both sides of all plant leaves. The amount of distilled water sprayed onto the control plants was provided with an equivalent volume of ethyl alcohol.

### 2.4. Assessment of Leaf Pigments, Chlorophyll Fluorescence, Gas Exchange, PSII Quantum Yield, and Photochemical Activity

Contents of carotenoids and chlorophylls were determined following the method in Arnon [44]. For extraction, a clean mortar and pestle, acetone (10 mL at 80% *v/v* for each sample), and 0.2 g of fresh tissue from the upper fourth leaf of the maize hybrids was used. After filtration, the optical densities of the filtrates (supernatants) were monitored at 663, 645, and 480 nm with a UV-120 spectrophotometer (Shimadzu Corp., Kyoto, Japan).

In the field, chlorophyll fluorescence was measured in the fresh upper fourth leaf of the maize hybrids using a fluorometer (FMS-2, portable, pulse-modulated, Hansatech, Norfolk, UK). The upper 4th leaf on each plant was subjected to light for 2 min until a constant rate of photosynthesis was reached. Steady-state fluorescence (Fs), maximum light-adaptive fluorescence (Fm), and minimum-adaptive fluorescence (F0) were measured [45]. *Fv/Fm* (maximum quantum yield of PSII) was calculated using Maxwell and Johnson formulae [46].

Using the fresh upper fourth leaf, the infrared gas analyzer apparatus (LCA-4 model, Anal. Dev. Co., Hoddesdon, England) was utilized to evaluate the rates of net photosynthesis (Pn), CO_2_ assimilation (A), and transpiration (E), as well as the conductance of the leafy stomata (gs). Measurements were repeated at 2-h intervals (7:00 a.m. to 5:00 p.m.) four times at 40, 50, 60, and 70 DAS. The photochemical activity was determined according to Jagendorf [47], with some modifications immersed in the method of Avron [48] using the Ferricyanide technique.

### 2.5. Assessment of Relative Content of Water (RWC), Stability Index of Cellular Membranes (MSI), Peroxidation of Lipids, Ion Leakage (EL), Soluble Sugars, and Proline

RWC was evaluated for maize hybrid leaves according to Osman and Rady [49] using 20 discs of 2 cm in diameter from a midrib-free fresh upper fourth leaf. To record discs’ fresh mass (FM) and turgid mass (TM), respectively, discs were weighed and then transferred to a dark location to be saturated by immersion in completely ion-free distilled water for 24 h. After dry blotting any water adhered, TM was taken. The discs’ dry mass (DM) was also taken after being dehydrated using an electric oven. To calculate the RWC, the following formula was applied:

RWC (%) = [(FM − DM)/(TM − DM)] × 100(1)

MSI was evaluated for maize hybrid leaves following the method detailed in Rady [50]. Two samples (0.2 g each) were taken from fresh upper fourth leaf tissue. Both samples were heated at 40 °C for 30 min and boiled at 100 °C for 10 min, respectively, after being immersed in test tubes with 10 mL of completely deionized distilled water. Using a conductivity bridge (Starlac Industries, Ambala, Haryana, India), solution electrical conductivity was recorded for both solutions (EC_1_ and EC_2_). To calculate the MSI, the following formula was applied:

MSI (%) = [1 − (EC_1_/EC_2_)] × 100 (2)

The total metallic ions infiltrated from hybridized maize leaf cells were evaluated following the method described by Rady [50]. Solution electrical conductivity of 20 leaf tissue discs was measured three times (EC_1_, EC_2_, and EC_3_). The three measurements were taken after immersing the discs in boiling tubes with 10 mL of completely deionized distilled water, after heating at 45–55 °C for 30 min and after boiling at 100 °C for 10 min, respectively. To calculate the EL, the following formula was applied:

EL (%) = [(EC_2_ − EC_1_)/EC_3_] × 100 (3)

Lipid peroxidation was determined using the fresh upper fourth leaf tissue of maize hybrids by assessing the level (µmole g^−1^) of malondialdehyde (MDA). Assessment of MDA was performed utilizing the extracts prepared as described in the Heath and Packer [51] method for H_2_O_2_ assessment. The calculations were done utilizing “0.155 × 10^−3^ M^−1^ cm^−1^”—the molar extinction coefficient to obtain MDA contents.

The method in Irigoyen et al. [52] was used to extract (using ethyl alcohol 96%, *v/v*), as well as evaluate, the level (mg g^−1^) of soluble sugars in the dry material of the upper fourth leaf tissue of maize hybrids. After boiling the reaction mixture consisting of a leafy extract (100 μL) + fresh anthrone reagent (3 mL; prepared with H_2_SO_4_, 72% *v/v*) for 10 min, it was cooled to record the absorbance reading at 625 nm using a spectrophotometer (a Bausch and Lomb-2000, USA).

The dry material (DW) of the upper fourth leaf tissue of maize hybrids was utilized to evaluate the free proline level (as μmol g^−1^ DW) by following the method described by Bates et al. [53]. Sulfosalicylic acid (3%, *w/v*) was used to extract proline from 0.5 g of dry sample; then, the extraction was centrifuged (10,000× *g*) for 10 min. A fresh acid–ninhydrin solution (2 mL) was added to the same volume of supernatant and incubated (90 °C) for 30 min. The reaction was cool-terminated before another extraction was performed with 5 mL of toluene. In the dark at room temperature, separation of the toluene and aqueous phases was achieved to assess the proline level colorimetrically in the toluene fraction at 520 nm.

### 2.6. Assessment of Enzymatic Antioxidants Activity

Using liquid N, midrib-free upper leaf samples were collected to obtain enzymatic extracts and assay the enzymatic antioxidant activities. The methods described in Chance and Maehly [54] were practiced to assay catalase (CAT) and peroxidase (POD) activities. CAT activity was read at 240 nm as changes in absorbance every 20 s. Every 0.01 absorbance value per min was specified as a unit of CAT activity. The method of guaiacol oxidation was followed for POD activity assaying by recording the absorbance value change read at 470 nm every 20 s. Every 0.01 absorbance value per min was specified as a unit of POD activity. The method detailed by Giannopolitis and Ries [55] was practiced to check the SOD activity. Each one unit activity is equal to a SOD enzyme amount, causing a 50% inhibition of the photochemical reduction of nitro blue tetrazolium (NBT). The activities of CAT, POD, and SOD enzymes were expressed on a unit mg^−1^ total soluble protein basis.

### 2.7. Agronomic Traits Measurements

Plant height was measured as the distance from the soil surface to the base of the tassel for ten plants per plot at physiological maturity (110 DAS). In each plot, the three central rows were harvested by hand at ground level to be sun-dried for 10 days and tied in bundles. The plants were then weighed to measure the biological yield and then converted into kg per ha. In each plot, 10 ears were selected randomly and separated to assess the number of rows and grains on each ear. All separated ears of each plot were shelled and then weighed for grain yield, which was calculated as kg ha^−1^. Thousand-grain sets were counted from shelled ears and weighed to measure the 1000-grain weight.

### 2.8. Water Productivity of Maize Grain Yield (CWP_g_) and Biological Yield (CWP_b_)

CWP_g_ and CWP_b_ were calculated as the grain yield (kg m^−3^) or biological yield (kg ha^−1^) ratio to crop evapotranspiration (ET, mm), [56,57,58], which was calculated following the equation of water balance [59]:

CWP (kg m^−3^) = Yield ÷ (ET × 10) (4)

ET = IW + P + Cr + Dp ± Rf ± ∆S(5)

IW = amount of irrigation water (mm), P = annual precipitation (rainfall; mm), Cr = capillary rise to plant root zone (mm), Dp = deep percolation (mm), Rf = surface runoff (mm), and ∆S = soil moisture change in the plant root zone (mm). When the groundwater table was 20 m below the ground surface, Cr was taken as zero. Dp and Rf were neglected due to using a drip irrigation system. Water content of soil was measured by the method based on oven drying for all experimental plots. Soil samples were taken at a depth of 0–30, 30–60 cm, and 60–90 cm at sowing and periodically at the main stages: seedling, heading, filling, and maturity during two growing seasons. The collected values were modified into a volumetric basis and multiplied by the soil depth of the soil sample and bulk density.

### 2.9. Statistical Analysis

Data of agronomical and physiological traits were subjected to ANOVA appropriate for a split-split plot design to test the effects of individual factors: irrigation regimes, foliar application, and maize hybrids, as well as their interactions, which were considered as fixed factors. The years, replications, and their interactions were considered random effects. Considering years and replications as random sources of error allowed broadening the effects of treatments across years and replications [60]. The mean differences among the irrigation regimes, foliar application, maize hybrids, and their interactions were separated by the LSD test at the *p* ≤ 0.05 significance level. Principal component analysis was implemented on averages of the evaluated physiological and agronomic traits to assess the relationship among them. The R software version 3.6.1 (https://www.r-project.org/) was used to perform the analyses.

## 3. Results

### 3.1. Leaf Photosynthetic Pigments, Photochemical Activity, and Photosynthetic Efficiency (Fv/Fm)

Moderate drought stress (MDS; 6000 m^3^ ha^−1^) and severe drought stress (SDS; 3000 m^3^ ha^−1^) significantly decreased the photosynthetic pigments (total chlorophyll and carotenoids), photochemical activity, and photosynthetic efficiency in all tested maize hybrids comparing with the well-watered (WW; 9000 m^3^ ha^−1^ as a control) conditions (Table 2). The adverse effects of SDS exceeded those of MDS and decreased the total chlorophyll, carotenoids, photochemical activity, and photosynthetic efficiency by 49.1%, 54.0%, 32.6%, and 25.5% in the same order compared with the WW plants. However, a foliar application with 24-epibrassinolide (EBR_24_) significantly improved all the above parameters in all drought-stressed hybrids when compared to the corresponding control group. In comparison with untreated plants, the EBR_24_ application increased the total chlorophyll by 20.6%, carotenoids by 18.6%, photochemical activity by 13.6%, and photosynthetic efficiency by 10.0%. The improved impact of EBR_24_ was more pronounced under MDS than under SDS. Maize hybrids exhibited variations in their responses to drought stress and EBR_24_ foliar application. Under the WW and MDS conditions, in general, the hybrid Giza-168 had the highest values of photosynthetic pigments and efficiency and photochemical activity, followed by Fine-276 and Giza-167, while the best values of these tested parameters were found in Fine-276, followed by Giza-167 and Giza-168, under SDS conditions. Generally, compared to the corresponding controls, the hybrid Giza-168 showed the highest contents of total chlorophylls (26.8%) and carotenoids (27.8%), photochemical activity (13.2%), and *Fv/Fm* (10.6%) as a response to EBR_24_ under MDS, while the hybrid Fine-276 displayed the highest response by 60.8%, 46.2%, 13.9%, and 13.8%, respectively, under SDS.

### 3.2. Gas Exchange and Lipid Peroxidation

The gas exchange criteria (i.e., rates of transpiration (Tr) and net photosynthesis (Pn), as well as stomatal conductance (gs)) were notably decreased, while the lipid peroxidation level (assessed as the level of malondialdehyde; MDA) was markedly elevated in all investigated hybrids of maize under MDS by 19.1%, 21.9%, 21.1%, and 29.6%, respectively, and SDS by 46.6%, 47.1%, 41.5%, and 62.4% compared with the WW conditions (Table 3). The decrease in gas exchange criteria and the increase in the MDA content were noteworthy with SDS compared to MDS. However, the EBR_24_ foliar application greatly reversed the trend above while significantly increasing all gas exchange criteria and significantly decreasing the MDA content in all drought-stressed hybrids compared to the corresponding control group. The EBR_24_ application increased the net photosynthesis rate, transpiration rate, and stomatal conductance by 22.0%, 17.9%, and 14.4%, respectively, while decreasing the MDA by 8.0% compared with untreated plants. The improved influence of EBR_24_ was more pronounced under SDS than under MDS. Maize hybrids displayed different responses to MDA and SDS. The highest values of gas exchange criteria and lowest value of MDA were observed with Giza-168 followed by Fine-276 under MDS, while, under SDS, Fine-276 collected the highest values of gas exchange criteria and lowest value of MDA content followed by Giza-167.

### 3.3. Relative Content of Water (RWC), Stability Index of Cell Membranes (MSI), and Leakage of Electrolytes (EL), as well as Soluble Sugars Content

The water uptake and leaf water content are strongly linked to the availability of soil water. The SDS impedes the plant’s osmotic system and leads to less water uptake, which leads to the wilting of plants and, finally, plant death. In this study, two levels of drought (MDS and SDS) significantly negatively affected the water status in all tested hybrids by decreasing the RWC by 20.9% and 44.0% and MSI by 26.3% and 51.9%, which were associated with an increased EL by 30.2% and 56.7% and soluble sugars content by 79.4% and 150.0% (Table 4). The trend of these results was more pronounced under SDS compared to MDS. However, the application of EBR_24_ significantly improved the RWC by 12.6% and MSI by 17.9%, which were associated with a considerable reduction in EL by 9.0% and a further elevation in the soluble sugars content by 12.2% in all the hybrids examined compared with the corresponding untreated controls. The best values of the RWC, MSI, and soluble sugar content and the lowest value of EL were obtained with Giza-168 and Fine-276 under MDS and SDS, respectively.

### 3.4. Free Proline Content and Antioxidant Enzymes Activity

When plants face drought stress, the activation processes begin to boost the plant’s defense system (nonenzymatic antioxidants coupled with enzymatic ones), leading to low molecular weight antioxidants and more production of antioxidative enzymes. The levels and activities of free proline, catalase (CAT), superoxide dismutase (SOD), and peroxidase (POD) were substantially elevated under the conditions of MDS by 113%, 75%, 104%, and 91% and SDS by 164%, 178%, 241%, and 238% compared to WW conditions in all investigated maize hybrids (Table 5). However, the supplementation of plant foliage with EBR_24_ markedly boosted the levels and activities of free proline, POD, CAT, and SOD by 5.1%, 17.9%, 16.0%, and 26.0%, respectively. The improvements in enzymatic and nonenzymatic activities were more pronounced under the SDS conditions in all hybrids. The highest values of free proline and all tested antioxidant enzymes were collected by Giza-168 and Fine-276 under MDS and SDS, respectively.

### 3.5. Yield and Yield-Contributing Traits

**Figure 1:**
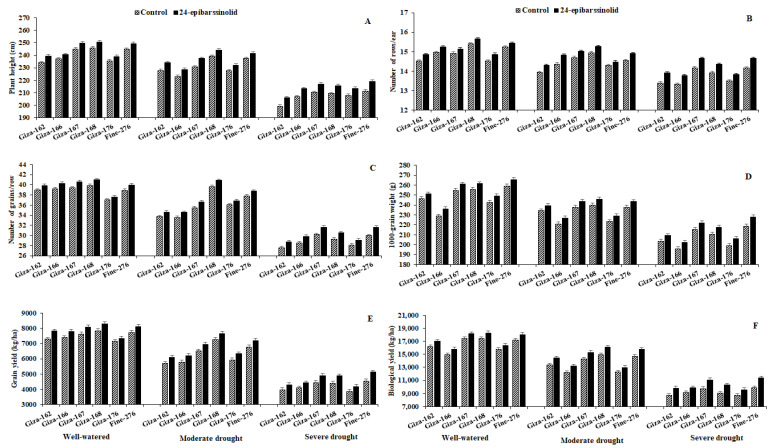
Influence of 24-epibrassinolide application on plant height (**A**), number of rows/ear (**B**), number of grains/row (**C**), 1000-grain weight (**D**), grain yield (**E**) and biological yield (**F**) of six maize hybrids grown under three irrigation regimes over the two growing seasons of 2018 and 2019. The bars on the top of the columns represent the LSD (*p* ≤ 0.05).

All measured agronomic traits: plant height, number of rows per ear, number of grains per ear, 1000-grain weight, grain yield/ha, and biological yield/ha were significantly decreased in all examined hybrids under MDS by 3.7%, 2.9%, 7.2%, 6.3%, 15.2%, and 16.3% and SDS by 13.1%, 7.3%, 24.9%, 16.1%, 42.5%, and 42.1% compared to the WW conditions (Figure 1). The reductions in the agronomic traits were more pronounced under SDS compared to MDS. Nevertheless, the application of EBR_24_ significantly enhanced all agronomic traits in all studied hybrids compared to the corresponding untreated controls. EBR_24_ application increased the plant height by 3.4%, number of rows per ear by 3.4%, number of grains per row by 4.2%, 1000-grain weight by 3.8%, grain yield/ha by 6.8%, and biological yield/ha by 7.6%. The responses to drought stress were proven by all hybrids with variations in their survival. Generally, Giza-168 showed the highest values for agronomic traits under the WW and MDS conditions, followed by Fine-276, then Giza-167. While under SDS, Fine-276 showed the highest values (especially for the biological yield by 15.2% and grain yield by 12.5%), followed by Giza-167, then Giza-168. In contrast, Giza-162, Giza-166, and Giza-176 showed the lowest agronomic performances, especially in light of the MDS and SDS.

### 3.6. Water Productivity of Grain Yield (CWP_g_) and Biological Yield (CWP_b_)

The results of the CWP_g_ and CWP_b_ as affected by the irrigation regimes, application of EBR_24_, and investigated hybrids are presented in Table 6. In general, the CWP_g_ varied from 0.73 to 1.35 kg m^−3^, and the CWP_b_ ranged from 1.53 to 3.0 kg m^−3^. Under the MDS and SDS conditions, the hybrids possessed higher CWP_g_ (on average, 0.96 and 1.17 kg m^−3^ in the same order) and CWP_b_ (on average, 2.08 and 2.58 kg m^−3^, respectively) than the WW condition (0.79 and 1.72 kg m^−3^ for CWP_g_ and CWP_b_, respectively). There is a higher CWP under IWD than the WW condition, because under available low water, the plants consumed water more efficiently and reduced the water loss. The application of EBR_24_ significantly improved the CWP_g_ by 7.6% (on average 1.01 kg m^−3^) and CWP_b_ by 8.7% (2.22 kg m^−3^) compared to the untreated controls (0.94 and 2.04 kg m^−3^ for CWP_g_ and CWP_b_, respectively). Additionally, the evaluated hybrids manifested different CWP_g_ and CWP_b_ under the irrigation regimes. The hybrid Giza-168 displayed the highest values under the MDS condition, followed by Fine-276, then Giza-167. Further, the best values under SDS were assigned for Fine-276, followed by Giza-167, then Giza-168.

### 3.7. Traits Interrelationship

**Figure 2:**
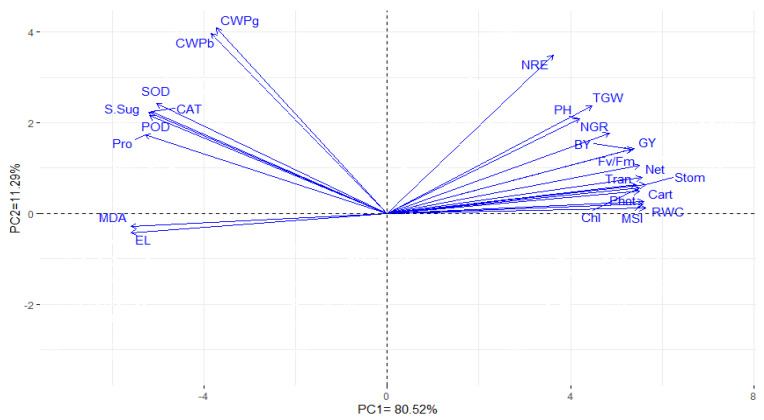
Biplot of the principal components analysis representing the relationship among the evaluated physiological and agronomic traits of six maize hybrids grown under three irrigation regimes over two growing seasons. Chl is total chlorophyll content, Car is the carotenoids content, Phot is the photochemical activity, *Fv/Fm* is the photosynthetic efficiency, Net is the net photosynthetic rate, Tran is the transpiration rate, Stom is the stomatal conductance, MDA is malondialdehyde, RWC is the relative water content, MSI is the membrane stability index, EL is the electrolyte leakage, S.Sug is soluble sugar, Pro is the proline content, POD is peroxidase, CAT is the catalase activity, SOD is the superoxide dismutase activity, PH is the plant height, NRE is the number of rows per ear, NGR is the number of grains per row, TGW is the 1000-grain weight, GY is the grain yield (kg ha^−1^), BY is the biological yield (kg ha^−1^), CWPg is the crop water productivity of the grain yield (kg m^−3^), and CWPb is the crop water productivity of the biological yield (kg m^−3^).

The associations among the evaluated agronomic and physiological traits were estimated based on the analysis of principal components. The first two principal components displayed most of the variance, about 91.81% (80.52% by PC1 and 11.29% by PC2); subsequently, they were used to construct the biplot (Figure 2). The traits represented by vectors with acute angles were revealed as robust positive associations, while those located with angles more than 90° showed negative associations. The traits evaluated in this study could be divided into three groups. The first group comprised the following traits: agronomic traits, total chlorophylls content, carotenoids content, photochemical activity, photosynthetic efficiency, rates of net photosynthesis and transpiration, relative content of water, conductance of leafy stomata, and stability index of the cell membranes. The second group consisted of CWP_g_ and CWP_b_; soluble sugar; the proline content; and the antioxidative enzyme activities (e.g., CAT, POD, and SOD), while the third group contained malondialdehyde and electrolyte leakage.

## 4. Discussion

The sustainable production of maize crops is currently facing a great problem of environmental degradation due to many issues, including irrigation water shortage (IWD). IWD is one of the major problems facing crop productions (including maize), especially in dry (arid and semi-arid) regions, which limits crop productivity. Improving IWD tolerance in maize through ecofriendly sustainable strategies is the key to securing foods for the growing human population [61].

The promotional influences of 24-epibrassinolide (EBR_24_) on plant growth under drought stress have been reported; however, little information is available on the EBR_24_-induced drought stress-conferring mechanisms for improving the growth of maize hybrids [40,41]. In this study, the potential improvements in maize hybrid yield-contributing traits under drought stress by improving the mechanisms in plant physio-biochemistry due to a foliar spray with EBR_24_ are discussed.

IWD reduced the yield-contributing components of all maize hybrids (e.g., plant height, row and grain numbers on each ear, 1000-grain weight, grains, and biological yield per ha) to varying degrees based on the tolerance or sensitivity of the maize hybrid (Figure 1). However, the foliar application of 5-μM EBR_24_ enabled maize plants to perform well under IWD stress, especially under moderate stress. EBR_24_ significantly reformed and awarded positive alterations in all indices of the plant morphology, physiology, and biochemistry and, consequently, the CWP and agronomic traits in all investigated maize hybrids growing under moderate (MDS) and severe drought stress (SDS) compared to the corresponding untreated controls. Photosynthetic traits, including photosynthetic pigments, are indicators of drought tolerance in plants. The results indicated that EBR_24_ enhanced the drought tolerance in maize hybrids by improving the chlorophyll and carotenoids contents, photochemical activity, and photosynthetic efficiency in the tested hybrids, especially under MDS (Table 2).

The promotional impacts of EBR_24_ on the yield-contributing traits observed in Figure 1 are potentially related to the improvements in the antioxidant system components (Table 5). These improvements are reflected in the reduction of membrane damage (reduced MDA content and EL) (Table 3 and Table 4) and in the protection of the photosynthetic apparatus (Table 2), which are attributed to the improvements in gas exchange, plant water status, and the osmo-protectant soluble sugars content (Table 3 and Table 4). This fact, coupled with the increase in chlorophyll content (Table 2) and MSI (Table 4) with the 5-µM EBR_24_ treatment, ensured the maintenance of the net photosynthetic rate (Table 3), reflecting an increase in the yield-contributing traits and final yields. Additionally, EBR_24_ promoted the transpiration rate and stomatal conductance under IWD stress conditions potentially due to how EBR_24_ acts in the stomatal closure in *Arabidopsis thaliana* in an abscisic acid (ABA)-independent manner [62]. Besides, EBR_24_ may act by modulating ABA-mediated stomatal closure both positively and negatively, depending on its concentration. In fact, the process of stomatal opening and closure is not only ABA-dependent. In IWD stress conditions, a dose-dependent action has also been reported for other hormones, such as cytokinins and auxins [62]. Therefore, it is possible that the dose-dependent action of EBR_24_ on stomatal behavior is due to its crosstalk with other plant hormones [32].

In the current study, due to oxidative damage to cell membranes under IWD stress, lipid peroxidation was identified as a level of malondialdehyde (MDA), an important biochemical marker of stress-inducing oxidative damage, as it minifies the production of the biomass, along with the plant’s acclimatization hypothesis [63,64]. Membrane lipid peroxidation is increased under drought stress, and as a consequence, the level of MDA is increased [65,66]. A low MDA level indicates a lower damage level to stressed cellular membranes, meaning that the plant is more stress-tolerant. When the peroxidase (POD) activity is associated with the level of membrane lipid peroxidation, a marked rise in the MDA level in stressed plants indicates an insufficient POD activity in the ROS collection to prevent damage to cellular membranes and minimize MDA production. In this study, the use of EBR_24_ made the plants able to avoid IWD stress and reduce their MDA levels compared with the untreated plants (Table 3), demonstrating the pivotal role of EBR_24_ in reducing lipid peroxidation and maintaining plasma membrane stability and structure under IWD stress. The lipid peroxidation reduction that occurred by EBR_24_ was connected with an increased enzymatic antioxidant activity, upregulating the membrane permeability in terms of the increased MSI (Table 4). These findings are consistent with those reported in [67,68].

The status of water in plants is highly sensitive to drought stress and is thus predominant in assessing a plant’s response to stress. IWD reduces the hydraulic conductivity of plant roots and water flow from root system to shoot system, reducing the leaf water content and closing the stomata to maintain leaf water [69,70]. A low RWC of plant leaves causes a toxic effect, which leads to physiological and metabolic changes and the inhibition of plant growth. However, the use of EBR_24_ markedly increased the leaf RWC and MSI in all IWD-stressed maize hybrids compared to the untreated controls (Table 4). This positive finding could result from the improvement of the transpiration rate in the treated stressed plants (Table 3). The same results were obtained by Shahid et al. [71] and Lima and Lobato [72]. EBR_24_ maintains the plant RWC under IWD stress by improving the water, pressure, and osmotic (solute) potentials due to the beneficial role of EBR_24_ in sustaining cellular membrane permeability and integrity under IWD stress conditions [73]. Besides, Rady [50] suggested that EBR_24_ affects the protein and/or enzyme biosynthesis to enhance the plant metabolism by improving the expression of specific genes [74].

In the current study, IWD considerably increased the leakage of electrolytes (EL). However, the use of EBR_24_ significantly reduced the EL in all the maize hybrids evaluated under two levels (MDS and SDS) of drought (Table 4). When the plants are exposed to drought, the leaf stomata are closed, causing a reduction in the fixation of CO_2_, while the transfer of electrons and the light reaction remain naturally. These conditions restrict the acceptance of electrons by NADP; thereby, oxygen can perform as an electron acceptor, resulting in the overproduction of ROS (e.g., O_2_^•−^, H_2_O_2_, and OH^−^), which peroxidize the cell membranes and increase the EL [75,76]. In this regard, Rady [50] reported a maximization of the EL and MDA in stressed plants. However, a follow-up treatment using 5-μM EBR_24_ decreased the lipid peroxidation and ionic leakage. Likewise, Shakirova et al. [30] and Mohammadi et al. [34] disclosed that the lowest MDA content in EBR_24_-treated plants exposed to SDS was associated with mitigating the stress-induced deleterious influences by enhancing the accumulation of osmolytes. This kept the cell membrane integrity, reduced the lipid peroxidation level, and produced various important metabolites. The use of EBR_24_ induced physio-biochemical alterations, including increasing the root system size, nonenzymatic antioxidant content, and enzyme activity [77].

In the present study, under IWD conditions, the soluble sugar content was significantly modified in all maize hybrids to contribute to osmotic modification and can, indirectly or directly, modify the gene expression implicated in plant metabolism and storage and defense functions [78,79]. Further, 5-μM EBR_24_ highly increased the soluble sugar content in all maize hybrids evaluated under the three irrigation regimes (Table 4). Like soluble sugars, the accumulation of free proline contributed to osmotic modification under IWD stress due to acclimatization to recompense for plant survival and, thus, helped in resisting drought stress [80]. Free proline enhances plant tolerance by the detoxification of ROS and may quench the singlet oxygen (^1^O_2_) in a physical manner or react directly with OH^−^ radicals [6]. Depending on the stress severity, the free cellular proline content is estimated to be approximately 20–80% of the total amino acid pool versus 5% under normal conditions, resulting from a decreased degradation and/or an increased biosynthesis of free proline in plants [81,82]. In this study, the total proline was increased by 5-μM EBR_24_, and the maximum concentration was observed under SDS (Table 5). Similarly, Talaat and Shawky [67] and Chen et al. [83] demonstrated the use of EBR_24_ initiates and increased proline biosynthesis in plant cells to enhance the plant defense system to avoid oxidative damage stimulated by IWD stress [4,34].

Under MDS, the CAT, SOD, and POD activities were increased and further increased under SDS conditions (Table 5). A strong correlation has been reported between oxidative stress tolerance and boosted enzymatic activities [84,85]. The plant tends to raise its antioxidant enzyme activities to withstand drought stress and eliminate ROS. These enzyme activities were markedly varied in the six maize hybrids assessed for their tolerance to IWD stress (Table 5). Moreover, the application of EBR_24_ boosted the CAT, POX, and SOD activities under IWD stress levels compared to the corresponding untreated control group (Table 5), which can be attributed to the EBR_24_ influence on transcription and/or translation of antioxidant genes [39,41,86].

Crop water productivity (CWP) refers to the association between crop productivity and the water amount used in crop production [56,57]. Ameliorating the crop water productivity is critical to producing more food using less water, particularly in arid and semi-arid environments, to preserve the limited irrigation water. The obtained results revealed that the application of EBR_24_ substantially increased the CWP_g_ and CWP_b_ by 9.9% and 12.4% under SDS and 6.6% and 7.4% under MDS compared to the untreated controls. The enhancement of CWP occurred through improving the photosynthetic efficiency, gas exchange indices, osmotic adjustment, water relations, and activities of antioxidant enzymes.

Identifying drought-tolerant maize hybrids is a pivotal approach to avoiding the destructive influences of drought stress, especially in arid environments, in light of the current climate changes. In the present study, the physiological parameters, CWP, and agronomic traits were used to assess the response of six hybrids of maize to IWD. Significantly, the examined hybrids demonstrated differences in their physiological and agronomic responses under IWD stress conditions. The hybrids introduced a significant alteration in the attributes of photosynthetic efficiency and gas exchange under three irrigation regimes (control; 9000 m^3^ water ha^−1^ versus 6000 and 3000 m^3^ water ha^−1^ applied as MDS and SDS, respectively). The hybrid pattern changed further under SDS compared to the MDS and well-watered conditions. The highest values of photosynthetic efficiency and gas exchange indices were assigned for Giza-168 under the MDS and well-watered conditions, followed by Fine-276, then Giza-167, while, under SDS, these indices exhibited the highest values by Fine-276, followed by Giza-167, then Giza-168 (Table 2 and Table 3). The rates of transpiration and gas exchange are related to the carbon uptake through opened stomata and the avoidance of dehydration, defined as the capability of plants to keep a high state of water. Therefore, these physiological behaviors helped the plants perform better under MDS and SDS (Figure 1).

Osmotic adjustment is a principal plant adaptive reaction to IWD stress at the cellular level. It is one of the components of turgor maintenance and dehydration avoidance and, therefore, has positive effects on the grain yield and related traits under IWD. In response to IWD stress, plants tend to accumulate inorganic and organic substances such as free proline, soluble sugars, and metallic ions to lessen the osmotic potential and boost the cell water retention [11,87]. Accordingly, the osmotic adjustment maintains a high RWC under a low water potential to meet transpiration upon request, sustains cellular turgor, promotes cell expansion, and, hence, the yield-forming processes [11,13,88]. In the current study, the Giza-167, Giza-168, and Fine-276 hybrids exhibited the highest free proline and soluble sugar contents under MDS and SDS compared to the other hybrids (Table 4 and Table 5). The large accumulation of free proline and soluble sugars contents provides an important adjusting role to postpone dehydration under unfavorable osmotic stress conditions and preserve a high RWC and MSI in these three hybrids (Table 4).

Under normal metabolism, ROS are normally produced in low levels in plant cells; however, they are produced excessively under adverse conditions, including IWD [89]. ROS production is controlled by the plant defense system, including CAT, SOD, POD, etc. and low-molecular-weight antioxidants [90]. POD can positively modify the levels of ROS through scavenging/consuming H_2_O_2_. Moreover, CAT and SOD can restrain, or at least minimize, OH^−^ radical generation [91,92]. In the current study, the ROS levels in the maize hybrids may be eliminated due to the significant improvement in CAT, POD, and SOD activities under IWD conditions (Table 5). In particular, Fine-276, Giza-167, and Giza-168 showed the highest antioxidant enzyme activities under MDS and SDS, which improved their agronomic performance and grain yield under IWD stress conditions.

The increment in gas exchange, photosynthetic efficiency, soluble sugar, proline contents, and antioxidant enzyme activities in maize hybrids may explain an increase in their agronomic traits under IWD stress conditions (Figure 1). Accordingly, Fine-276 displayed the greatest ability to accumulate biomass in the shoot, followed by Giza-167 and Giza-168, as shown by the largest plant height, grain yield, and contributing traits. These hybrids demonstrated a mechanism to withstand dehydration by enhancing the efficiency of photosynthesis, gas exchange, osmotic adjustment, water relations, and enzymatic antioxidant activities. Furthermore, the drought-tolerant hybrids exhibited more grain and biological yields with higher CWP_g_ and CWP_b_ (Table 6) compared to the drought-sensitive ones. The hybrids Giza-168, Fine-276, and Giza-167 displayed better CWP values associated with significantly greater growth and productivity—in particular, at low water amounts—compared to the sensitive ones. Therefore, using these drought-tolerant hybrids is preferred to improve the CWP and increase the grain and biological yields principally in arid environments [93,94,95,96].

The constituent plant traits have a crucial function in water use, dehydration avoidance, and producing an acceptable grain yield under IWD [11]. Consequently, the yield potential can be defined as the traits that can boost the yield under IWD during the growth stages. The traits that confer drought tolerance can be divided into two types: firstly, improving the crop yield during water supply, and the second, which contributes to the survival of the plant during a very limited capacity of the soil to hold water [13,97].

Assessing the interrelationships between plant traits can provide useful information for screening maize hybrids under low available water conditions. A biplot of principal components is an appropriate statistical method for understanding the interrelationships among evaluated traits, which is estimated by the angle size of the trait vectors (Figure 2). The results reflected that the agronomic traits were positively associated with the total chlorophyll and carotenoid contents, photochemical activity, rates of net photosynthesis and transpiration, photosynthetic efficiency, conductance of leafy stomata, RWC, and MSI. From this standpoint, it could be speculated that the high values of these physiological traits could illustrate more grain yields and contributing traits. Otherwise, the CWP_g_ and CWP_b_ proved to have highly positive associations with the levels of soluble sugars and free proline and activities of POD, CAT, and SOD. Additionally, the agronomic traits displayed highly negative associations with malondialdehyde and the electrolyte leakage. These results are in consonance with previous studies that have demonstrated the importance of physiological parameters as indicators for grain yield under abiotic stress [98,99,100,101,102]. According to these findings, it is important to detect certain physiological traits that have a positive association with yield-related traits or CWP under drought stress.

## 5. Conclusions

Drought stress decreased the growth and yield-contributing traits in maize hybrids to varying degrees. It also reduced the photosynthetic efficiency, gas exchange parameters, membrane stability index, plant water status, and increased the lipid peroxidation and electrolyte leakage. These adverse impacts were alleviated by the foliar-used 24-epibrassinolide (5 µM) by promoting the parameters mentioned above due to the decrease in the membrane electrolyte leakage and lipid peroxidation. These positive results were obtained via the better upregulation and activity of osmo-protectants and different antioxidant system components under drought stress. Additionally, the improved water status of IWD-stressed maize hybrids by 5-µM 24-epibrassinolide enhanced the crop water productivity. Certain physiological traits contributed to highly positive associations with yield-related traits or crop water productivity under drought stress. In addition, the increased activation of the plants’ defense systems by 24-epibrassinolide resulted in a greater adaptation of maize plants to drought stress. The 24-epibrassinolide applications could introduce a simple strategy in maize production in dry regions; however, further studies are needed to assess the efficiency of 24-epibrassinolide under different open field conditions. The use of a surfactant that is retained on the leaf surface and stays in the spraying solution of 24-epibrassinolide for a longer period is likely to increase the yield response under controlled and different open field conditions. In the future, it is commercially possible to implement the use of 24-epibrassinolide under open field conditions, but the provided 24-epibrassinolide should be added to an appropriate surfactant, and the spraying solution must be prepared and used immediately in the early morning so that it is a beneficial agronomic practice.

## Figures and Tables

**Table 1:** Monthly average minimum (Tmin) and maximum (Tmax) temperatures, growing degree days (GDD), and total precipitation (Perc.) in the 2018 and 2019 growing seasons of maize and 35 years of monthly averages (1985 to 2019).

	2018				2019				35 Years Average		
Month	Tmin	Tmax	GDD ^†^	Perc.	Tmin	Tmax	GDD ^†^	Perc.	Tmin	Tmax	Perc.
	°			mm	°C			mm	°C		mm
May	18.20	35.60	539.1	0.15	17.80	36.20	536.9	0.40	16.82	34.53	0.23
June	21.20	37.80	584.7	0.00	21.60	38.30	598.3	0.00	19.63	37.91	0.30
July	22.40	39.00	641.3	0.35	22.40	39.30	644.9	0.00	21.30	39.10	0.22
August	22.60	38.40	635.7	0.00	22.70	39.00	646.7	0.00	21.76	38.81	0.02
September	21.00	36.30	560.1	0.00	20.70	36.00	531.8	0.00	20.05	36.21	0.30

^†^ Growing degree days was calculated from the daily minimum and maximum temperatures based on a 10 °C base temperature and accumulated for each month.

**Table 2:** Impact of 24-epibrassinolide (EBR_24_) application on the total chlorophyll content, carotenoids, photochemical activity, and photosynthetic efficiency (*Fv/Fm*) of six maize hybrids grown under three irrigation regimes over the two growing seasons of 2018 and 2019.

Irrigation	Hybrids	Total Chlorophyll (mg g^−1^ FW)			Carotenoids (mg g^−1^ FW)			Photochemical Activity			*Fv/Fm*		
		Cont. ^†^	EBR_24_	Mean *	Cont.	EBR_24_	Mean	Cont.	EBR_24_	Mean	Cont.	EBR_24_	Mean
Well-watered	Giza-162	2.69	2.86	2.77 ^d^	0.97	1.10	1.03 ^d^	41.23	47.63	44.43 ^d^	0.817	0.900	0.858 ^d^
	Giza-166	2.68	2.81	2.74 ^e^	0.95	1.04	1.00 ^e^	40.73	47.23	43.98 ^e^	0.810	0.880	0.845 ^e^
	Giza-167	2.69	2.97	2.83 ^c^	0.97	1.14	1.06 ^c^	41.63	47.97	44.80 ^c^	0.827	0.920	0.873 ^c^
	Giza-168	2.73	3.14	2.94 ^a^	1.01	1.21	1.11 ^a^	43.50	48.87	46.18 ^a^	0.837	0.950	0.893 ^a^
	Giza-176	2.66	2.75	2.71 ^f^	0.94	1.02	0.98 ^f^	40.21	46.10	43.15 ^f^	0.807	0.877	0.842 ^e^
	Fine-276	2.71	3.04	2.87 ^b^	0.99	1.18	1.09 ^b^	42.73	48.30	45.52 ^b^	0.833	0.937	0.885 ^b^
	Mean	2.69 ^B^	2.93 ^A^	***2.81 ^A^***	0.97 ^B^	1.12 ^A^	***1.04 ^A^***	41.67 ^B^	47.68 ^A^	***44.68 ^A^***	0.822 ^B^	0.911 ^A^	***0.866 ^A^***
Moderate drought	Giza-162	1.98	2.34	2.16 ^e^	0.66	0.73	0.70 ^e^	33.70	37.27	35.48 ^c^	0.707	0.763	0.735 ^cd^
	Giza-166	1.99	2.46	2.23 ^d^	0.67	0.76	0.72 ^d^	34.20	37.60	35.90 ^b^	0.710	0.763	0.737 ^cd^
	Giza-167	2.02	2.55	2.29 ^c^	0.68	0.81	0.75 ^c^	33.97	38.10	36.03 ^b^	0.713	0.770	0.742 ^c^
	Giza-168	2.09	2.65	2.37 ^a^	0.71	0.90	0.81 ^a^	34.33	38.87	36.60 ^a^	0.727	0.803	0.765 ^a^
	Giza-176	1.95	2.19	2.07 ^f^	0.66	0.72	0.69 ^e^	33.27	36.80	35.03 ^d^	0.703	0.760	0.732 ^d^
	Fine-276	2.05	2.60	2.33 ^b^	0.69	0.87	0.78 ^b^	33.43	38.27	35.85 ^b^	0.720	0.783	0.752 ^b^
	Mean	2.01 ^B^	2.47 ^A^	***2.24 ^B^***	0.68 ^B^	0.80 ^A^	***0.74 ^B^***	33.82 ^B^	37.82 ^A^	***35.82 ^B^***	0.713 ^B^	0.774 ^A^	***0.744 ^B^***
Severe drought	Giza-162	1.16	1.67	1.41 ^d^	0.42	0.52	0.47 ^d^	27.63	32.50	30.07 ^c^	0.613	0.663	0.638 ^d^
	Giza-166	1.14	1.54	1.34 ^e^	0.41	0.49	0.45 ^e^	27.33	31.43	29.38 ^d^	0.603	0.663	0.633 ^de^
	Giza-167	1.19	1.87	1.53 ^b^	0.43	0.59	0.51 ^b^	28.47	32.60	30.53 ^b^	0.620	0.693	0.657 ^b^
	Giza-168	1.17	1.76	1.47 ^c^	0.43	0.55	0.49 ^c^	27.97	32.23	30.10 ^c^	0.617	0.680	0.648 ^c^
	Giza-176	1.13	1.39	1.26 ^f^	0.40	0.45	0.43 ^f^	28.07	31.27	29.67 ^d^	0.597	0.657	0.627 ^e^
	Fine-276	1.21	1.94	1.57 ^a^	0.43	0.63	0.53 ^a^	28.97	33.00	30.98 ^a^	0.627	0.713	0.670 ^a^
	Mean	1.17 ^B^	1.69 ^A^	***1.43 ^C^***	0.42 ^B^	0.54 ^A^	***0.48 ^C^***	28.07 ^B^	32.17 ^A^	***30.12 ^C^***	0.613 ^B^	0.678 ^A^	***0.646 ^C^***
	**Mean (F)**	**1.96 ^B^**	**2.36 ^A^**		**0.69 ^B^**	**0.82 ^A^**		**34.52 ^B^**	**39.22 ^A^**		**0.716 ^B^**	**0.788 ^A^**	
ANOVA	**df**	***p*-value of the main effects and their interactions**											
Irrigation (I)	2	<0.001			<0.001			<0.001			<0.001		
Foliar (F)	1	<0.001			<0.001			<0.001			<0.001		
Hybrids (H)	5	<0.001			<0.001			<0.001			<0.001		
I × F	2	<0.001			<0.001			<0.001			<0.001		
I × H	10	<0.001			<0.001			0.001			<0.001		
F × H	5	<0.001			<0.001			<0.001			<0.001		
I × F × H	10	<0.001			<0.001			<0.001			<0.001		

^†^ Cont. is the control of which distilled water provided with the equivalent amount of ethyl alcohol was sprayed for control plants instead of the EBR_24_ solution. * Means followed by different letters in the same direction differ significantly by LSD (*p* < 0.05), bold and italic means belong to the three irrigation regimes, and bold and not italic means belong to the foliar applications.

**Table 3:** Impact of 24-epibrassinolide (EBR_24_) application on the net photosynthetic rate, transpiration rate, stomatal conductance, and malondialdehyde (MDA) of six maize hybrids grown under three irrigation regimes over the two growing seasons of 2018 and 2019.

Irrigation	Hybrids	Net Photosynthetic Rate			Transpiration Rate			Stomatal Conductance			MDA (µmol g^−1^ FW)		
		Cont. ^†^	EBR_24_	Mean *	Cont.	EBR_24_	Mean	Cont.	EBR_24_	Mean	Cont.	EBR_24_	Mean
Well-watered	Giza-162	9.20	11.42	10.31 ^d^	4.97	7.63	6.30 ^c^	0.627	0.710	0.668 ^d^	45.30	42.10	43.70 ^c^
	Giza-166	9.11	11.36	10.23 ^d^	6.91	7.55	7.23 ^ab^	0.620	0.690	0.655 ^e^	46.00	42.33	44.17 ^b^
	Giza-167	9.37	11.54	10.45 ^c^	6.99	7.74	7.37 ^ab^	0.637	0.730	0.683 ^c^	43.97	41.07	42.52 ^d^
	Giza-168	9.67	12.33	11.00 ^a^	7.15	7.95	7.55 ^a^	0.647	0.760	0.703 ^a^	42.27	39.33	40.80 ^f^
	Giza-176	8.99	10.51	9.75 ^e^	6.84	7.45	7.15 ^b^	0.617	0.687	0.652 ^e^	46.40	42.97	44.68 ^a^
	Fine-276	9.47	11.90	10.69 ^b^	7.02	7.86	7.44 ^ab^	0.643	0.77	0.695 ^b^	43.20	39.77	41.48 ^e^
	Mean	9.30 ^B^	11.51 ^A^	***10.41 ^A^***	6.65 ^B^	7.70 ^A^	***7.17 ^A^***	0.632 ^B^	0.721 ^A^	***0.676 ^A^***	44.52 ^A^	41.26 ^B^	***42.89 ^C^***
Moderate drought	Giza-162	7.24	8.53	7.89 ^c^	5.53	6.09	5.81 ^ab^	0.497	0.553	0.525 ^cd^	59.97	54.17	57.07 ^b^
	Giza-166	7.86	8.58	8.22 ^b^	5.29	6.19	5.74 ^ab^	0.500	0.553	0.527 ^c^	58.53	54.57	56.55 ^c^
	Giza-167	7.72	8.70	8.21 ^b^	5.33	6.31	5.82 ^ab^	0.503	0.560	0.532 ^c^	57.00	51.37	54.18 ^d^
	Giza-168	7.91	8.97	8.44 ^a^	5.43	6.52	5.98 ^a^	0.517	0.593	0.555 ^a^	55.87	50.13	53.00 ^e^
	Giza-176	7.10	8.20	7.65 ^d^	5.11	5.98	5.54 ^b^	0.493	0.550	0.522 ^d^	60.63	55.70	58.17 ^a^
	Fine-276	7.81	8.87	8.34 ^a^	5.39	6.45	5.92 ^ab^	0.510	0.573	0.542 ^b^	57.03	51.93	54.48 ^d^
	Mean	7.60 ^B^	8.64 ^A^	***8.12 ^B^***	5.35 ^B^	6.26 ^A^	***5.80 ^B^***	0.503 ^B^	0.564 ^A^	***0.534 ^B^***	58.17 ^A^	52.98 ^B^	***55.58 ^B^***
Severe drought	Giza-162	4.74	6.20	5.47 ^c^	3.21	4.15	3.68 ^bc^	0.363	0.413	0.388 ^d^	72.00	66.50	69.25 ^c^
	Giza-166	4.63	6.02	5.33 ^d^	4.02	4.13	4.08 ^a^	0.353	0.413	0.383 ^de^	76.07	70.27	73.17 ^b^
	Giza-167	4.86	6.47	5.67 ^b^	3.42	4.40	3.91 ^ab^	0.370	0.443	0.407 ^b^	68.87	63.33	66.10 ^e^
	Giza-168	4.82	6.37	5.60 ^b^	3.36	4.29	3.83 ^ab^	0.367	0.430	0.398 ^c^	71.43	65.37	68.40 ^d^
	Giza-176	4.51	5.84	5.17 ^e^	3.08	3.83	3.46 ^c^	0.347	0.407	0.377 ^e^	78.40	74.03	76.22 ^a^
	Fine-276	4.94	6.69	5.82 ^a^	3.49	4.56	4.02 ^ab^	0.377	0.463	0.420 ^a^	67.58	62.10	64.84 ^f^
	Mean	4.75 ^B^	6.27 ^A^	***5.51 ^C^***	3.43 ^B^	4.23 ^A^	***3.83 ^C^***	0.363 ^B^	0.428 ^A^	***0.396 ^C^***	72.39 ^A^	66.93 ^B^	***69.66 ^A^***
	**Mean (F)**	**7.22 ^B^**	**8.81 ^A^**		**5.14 ^B^**	**6.06 ^A^**		**0.499 ^B^**	**0.571 ^A^**		**58.36 ^A^**	**53.72 ^B^**	
ANOVA	**df**	***p*-value of the main effects and their interactions**											
Irrigation (I)	2	<0.001			<0.001			<0.001			<0.001		
Foliar (F)	1	<0.001			<0.001			<0.001			<0.001		
Hybrids (H)	5	<0.001			0.001			<0.001			<0.001		
I × F	2	<0.001			0.011			<0.001			<0.001		
I × H	10	0.005			0.028			0.002			0.004		
F × H	5	<0.001			0.045			<0.001			0.038		
I × F × H	10	<0.001			0.031			<0.001			0.002		

^†^ Cont. is the control of which distilled water provided with the equivalent amount of ethyl alcohol was sprayed for control plants instead of the EBR_24_ solution. * Means followed by different letters in the same direction differ significantly by LSD (*p* < 0.05), bold and italic means belong to the three irrigation regimes, and bold and not italic means belong to the foliar applications.

**Table 4:** Impact of the 24-epibrassinolide (EBR_24_) application on the relative water content (RWC), membrane stability index (MSI), electrolyte leakage (EL), and soluble sugars content of six maize hybrids grown under three irrigation regimes over the two growing seasons of 2018 and 2019.

Irrigation	Hybrids	RWC %			MSI %			EL (%)			Soluble Sugars (mg g^−1^ DW)		
		Cont. ^†^	EBR_24_	Mean *	Cont.	EBR_24_	Mean	Cont.	EBR_24_	Mean	Cont.	EBR_24_	Mean
Well-watered	Giza-162	81.27	85.93	83.60 ^c^	76.97	82.03	79.50 ^c^	21.73	19.23	20.48 ^c^	18.73	20.50	19.62 ^d^
	Giza-166	80.77	87.37	84.07 ^c^	76.27	80.33	78.30 ^d^	22.67	19.57	21.12 ^b^	18.07	20.27	19.17 ^e^
	Giza-167	82.23	87.53	84.88 ^b^	77.77	83.90	80.83 ^b^	21.40	19.37	20.38 ^c^	19.30	21.23	20.27 ^c^
	Giza-168	83.30	91.63	87.47 ^a^	79.37	86.07	82.72 ^a^	18.90	18.17	18.53 ^e^	20.43	23.10	21.77 ^a^
	Giza-176	80.42	85.37	82.89 ^d^	75.70	78.13	76.92 ^e^	23.10	20.27	21.68 ^a^	17.57	19.17	18.37 ^f^
	Fine-276	81.30	88.77	85.03 ^b^	77.27	84.90	81.08 ^b^	20.67	18.63	19.65 ^d^	19.70	22.10	20.90 ^b^
	Mean	81.55 ^B^	87.77 ^A^	***84.66 ^A^***	77.22 ^B^	82.56 ^A^	***79.89 ^A^***	21.41 ^A^	19.21 ^B^	***20.31 ^C^***	18.97 ^B^	21.06 ^A^	***20.01 ^C^***
Moderate drought	Giza-162	61.73	68.20	64.97 ^d^	50.80	61.77	56.28 ^e^	28.30	26.07	27.18 ^ab^	31.43	36.73	34.08 ^e^
	Giza-166	61.97	70.87	66.42 ^c^	51.43	63.67	57.55 ^d^	28.10	26.03	27.07 ^b^	33.33	38.00	35.67 ^d^
	Giza-167	62.90	73.53	68.22 ^b^	52.97	67.30	60.13 ^c^	27.17	24.73	25.95 ^c^	34.33	39.07	36.70 ^c^
	Giza-168	64.40	76.17	70.28 ^a^	54.93	70.90	62.92 ^a^	26.10	24.10	25.10 ^d^	36.57	40.97	38.77 ^a^
	Giza-176	61.10	65.27	63.18 ^e^	49.97	60.43	55.20 ^f^	28.33	26.83	27.58 ^a^	30.77	35.10	32.93 ^f^
	Fine-276	63.60	74.00	68.80 ^b^	53.80	68.73	61.27 ^b^	26.80	24.67	25.73 ^c^	35.10	39.43	37.27 ^b^
	Mean	62.62 ^B^	71.34 ^A^	***66.98 ^B^***	52.32 ^B^	65.47 ^A^	***58.89 ^B^***	27.47 ^A^	25.41 ^B^	***26.44 ^B^***	33.59 ^B^	38.22 ^A^	***35.90 ^B^***
Severe drought	Giza-162	42.43	49.99	46.21 ^d^	31.70	43.00	37.35 ^d^	34.07	30.20	32.13 ^b^	46.47	51.97	49.22 ^d^
	Giza-166	41.57	48.43	45.00 ^e^	30.47	40.73	35.60 ^e^	34.00	31.23	32.62 ^a^	46.33	50.87	48.60 ^e^
	Giza-167	43.63	54.73	49.18 ^b^	35.13	47.07	41.10 ^b^	32.17	29.63	30.90 ^d^	48.33	54.70	51.52 ^b^
	Giza-168	43.77	52.90	48.33 ^c^	33.80	44.77	39.28 ^c^	33.27	29.97	31.62 ^c^	47.40	54.07	50.73 ^c^
	Giza-176	40.55	47.37	43.96 ^f^	29.93	38.37	34.15 ^f^	34.10	31.43	32.77 ^a^	45.37	49.33	47.35 ^f^
	Fine-276	46.57	56.87	51.72 ^a^	37.90	48.73	43.32 ^a^	32.63	29.10	30.87 ^d^	49.87	55.77	52.82 ^a^
	Mean	43.09 ^B^	51.71 ^A^	***47.40 ^C^***	33.16 ^B^	43.78 ^A^	***38.47 ^C^***	33.37 ^A^	30.26 ^B^	***31.82 ^A^***	47.29 ^B^	52.78 ^A^	***50.04 ^A^***
	**Mean (F)**	**62.42 ^B^**	**70.27 ^A^**		**54.23 ^B^**	**63.94 ^A^**		**27.42 ^A^**	**24.96 ^B^**		**33.28 ^A^**	**37.35 ^B^**	
ANOVA	**df**	***p*-value of the main effects and their interactions**											
Irrigation (I)	2	<0.001			<0.001			<0.001			<0.001		
Foliar (F)	1	<0.001			<0.001			<0.001			<0.001		
Hybrids (H)	5	<0.001			<0.001			<0.001			<0.001		
I × F	2	<0.001			<0.001			<0.001			<0.001		
I × H	10	0.003			0.002			0.011			<0.001		
F × H	5	<0.001			<0.001			0.058			<0.001		
I × F × H	10	<0.001			<0.001			<0.001			<0.001		

^†^ Cont. is the control of which distilled water provided with the equivalent amount of ethyl alcohol was sprayed for control plants instead of the EBR_24_ solution. * Means followed by different letters in the same direction differ significantly by LSD (*p* < 0.05), bold and italic means belong to the three irrigation regimes, and bold and not italic means belong to the foliar applications.

**Table 5:** Impact of 24-epibrassinolide (EBR_24_) application on the proline content, peroxidase (POD), catalase activity (CAT), and superoxide dismutase activity (SOD) of six maize hybrids grown under three irrigation regimes over the two growing seasons of 2018 and 2019.

Irrigation	Hybrids	Proline (µmol g^−1^ DW)			POD (Unit mg^−1^ Protein)			CAT (Unit mg^−1^ Protein)			SOD (Unit mg^−1^ Protein)		
		Cont. ^†^	EBR_24_	Mean *	Cont.	EBR_24_	Mean	Cont.	EBR_24_	Mean	Cont.	EBR_24_	Mean
Well-watered	Giza-162	62.07	64.60	63.33 ^bc^	8.06	9.79	8.93 ^b^	4.33	5.74	5.03 ^d^	2.62	3.93	3.28 ^cd^
	Giza-166	60.93	63.60	62.27 ^c^	7.23	9.03	8.13 ^c^	3.93	5.23	4.58 ^e^	2.35	3.75	3.05 ^d^
	Giza-167	63.30	64.67	63.98 ^bc^	8.38	9.87	9.13 ^b^	4.74	5.88	5.31 ^c^	2.87	4.23	3.55 ^bc^
	Giza-168	66.20	69.03	67.62 ^a^	9.19	10.90	10.05 ^a^	5.28	6.59	5.94 ^a^	3.38	4.93	4.15 ^a^
	Giza-176	60.53	62.80	61.67 ^c^	6.93	7.80	7.36 ^d^	3.41	4.66	4.03 ^f^	2.17	3.51	2.84 ^d^
	Fine-276	64.40	66.70	65.55 ^ab^	9.04	10.34	9.69 ^a^	5.12	6.57	5.85 ^b^	3.06	4.80	3.93 ^ab^
	Mean	62.91 ^B^	65.23 ^A^	***64.07 ^C^***	8.14 ^B^	9.62 ^A^	***8.88 ^C^***	4.47 ^B^	5.78 ^A^	***5.12 ^C^***	2.74 ^B^	4.19 ^A^	***3.47 ^C^***
Moderate drought	Giza-162	131.63	134.30	132.97 ^c^	14.29	17.70	15.99 ^e^	7.80	9.05	8.42 ^e^	5.47	7.44	6.45 ^d^
	Giza-166	134.30	138.07	136.18 ^b^	14.79	18.47	16.63 ^d^	7.99	9.10	8.55 ^d^	5.91	7.90	6.90 ^bc^
	Giza-167	134.67	139.60	137.13 ^b^	15.17	18.94	17.06 ^c^	8.43	9.94	9.19 ^c^	6.17	8.10	7.14 ^b^
	Giza-168	137.70	142.80	140.25 ^a^	17.70	19.86	18.78 ^a^	9.03	10.85	9.94 ^a^	6.87	8.86	7.87 ^a^
	Giza-176	130.63	134.63	132.63 ^c^	13.31	17.47	15.39 ^f^	7.32	8.44	7.88 ^f^	6.14	6.84	6.49 ^c^
	Fine-276	135.60	140.87	138.23 ^ab^	16.28	19.13	17.71 ^b^	8.77	10.55	9.66 ^b^	6.51	8.70	7.61 ^a^
	Mean	134.09 ^B^	138.38 ^A^	***136.23 ^B^***	15.26 ^B^	18.60 ^A^	***16.93 ^B^***	8.22 ^B^	9.65 ^A^	***8.94 ^B^***	6.18 ^B^	7.97 ^A^	***7.08 ^B^***
Severe drought	Giza-162	163.67	174.10	168.88 ^b^	27.43	31.77	29.60 ^d^	13.25	14.33	13.79 ^d^	10.77	12.50	11.64 ^bc^
	Giza-166	162.40	172.60	167.50 ^bc^	26.74	31.17	28.95 ^e^	12.98	14.41	13.69 ^e^	10.54	12.25	11.39 ^c^
	Giza-167	167.50	177.57	172.53 ^a^	28.95	33.24	31.10 ^b^	14.01	15.63	14.82 ^b^	11.24	13.62	12.43 ^a^
	Giza-168	166.53	177.17	171.85 ^a^	28.24	32.66	30.45 ^c^	13.66	15.04	14.35 ^c^	10.95	12.72	11.83 ^b^
	Giza-176	160.57	169.93	165.25 ^c^	25.77	30.45	28.11 ^f^	12.73	14.35	13.54 ^f^	10.21	11.69	10.95 ^d^
	Fine-276	160.60	179.37	169.98 ^ab^	29.90	33.93	31.92 ^a^	14.36	15.95	15.16 ^a^	11.55	13.92	12.74 ^a^
	Mean	163.54 ^B^	175.12 ^A^	***169.33 ^A^***	27.84 ^B^	32.20 ^A^	***30.02 ^A^***	13.50 ^B^	14.95 ^A^	***14.22 ^A^***	10.88 ^B^	12.78 ^A^	***11.83 ^A^***
	**Mean (F)**	**120.18 ^B^**	**126.24 ^A^**		**17.08 ^B^**	**20.14 ^A^**		**8.73 ^B^**	**10.13 ^A^**		**6.60 ^B^**	**8.32 ^A^**	
ANOVA	**df**	***p*-value of the main effects and their interactions**											
Irrigation (I)	2	<0.001			<0.001			<0.001			<0.001		
Foliar (F)	1	<0.001			<0.001			<0.001			<0.001		
Hybrids (H)	5	<0.001			<0.001			<0.001			<0.001		
I × F	2	<0.001			<0.001			0.011			<0.001		
I × H	10	0.003			0.012			<0.001			0.031		
F × H	5	<0.001			0.011			<0.001			<0.001		
I × F × H	10	<0.001			0.001			<0.001			0.001		

^†^ Cont. is the control of which distilled water provided with the equivalent amount of ethyl alcohol was sprayed for control plants instead of the EBR_24_ solution. * Means followed by different letters in the same direction differ significantly by LSD (*p* < 0.05), bold and italic means belong to the three irrigation regimes, and bold and not italic means belong to the foliar applications.

**Table 6:** Impact of 24-epibrassinolide (EBR_24_) application on the crop water productivity of grain yield (CWP_g_, kg m^−3^) and biological yield (CWP_b_, kg m^−3^) of six maize hybrids grown under three irrigation regimes over the two growing seasons of 2018 and 2019.

Irrigation	Hybrids	Crop Water Productivity of Grain Yield (CWP_g_)			Crop Water Productivity of Biological Yield (CWP_b_)		
		Cont. ^†^	EBR_24_	Mean *	Cont.	EBR_24_	Mean
Well-watered	Giza-162	0.744	0.798	0.771 ^c^	1.651	1.738	1.695 ^c^
	Giza-166	0.755	0.796	0.776 ^c^	1.526	1.613	1.570 ^e^
	Giza-167	0.777	0.825	0.801 ^b^	1.776	1.858	1.817 ^ab^
	Giza-168	0.799	0.846	0.823 ^a^	1.778	1.869	1.824 ^a^
	Giza-176	0.729	0.750	0.739 ^d^	1.608	1.674	1.641 ^d^
	Fine-276	0.790	0.830	0.810 ^b^	1.752	1.843	1.797 ^b^
	Mean	0.766 ^B^	0.807 ^A^	***0.786 ^C^***	1.682 ^B^	1.766 ^A^	***1.724 ^C^***
Moderate drought	Giza-162	0.838	0.898	0.868 ^f^	1.971	2.130	2.051 ^d^
	Giza-166	0.852	0.915	0.883 ^e^	1.799	1.938	1.868 ^e^
	Giza-167	0.958	1.023	0.990 ^c^	2.102	2.254	2.178 ^c^
	Giza-168	1.069	1.128	1.099 ^a^	2.198	2.375	2.287 ^a^
	Giza-176	0.873	0.931	0.902 ^d^	1.807	1.911	1.859 ^e^
	Fine-276	0.997	1.058	1.028 ^b^	2.160	2.319	2.240 ^b^
	Mean	0.931 ^B^	0.992 ^A^	***0.962 ^B^***	2.006 ^B^	2.155 ^A^	***2.080 ^B^***
Severe drought	Giza-162	1.047	1.134	1.091 ^d^	2.298	2.593	2.445 ^e^
	Giza-166	1.080	1.164	1.122 ^c^	2.402	2.604	2.503 ^d^
	Giza-167	1.169	1.294	1.231 ^b^	2.575	2.920	2.747 ^b^
	Giza-168	1.160	1.286	1.223 ^b^	2.379	2.714	2.547 ^c^
	Giza-176	1.017	1.107	1.062 ^e^	2.302	2.531	2.416 ^f^
	Fine-276	1.200	1.351	1.275 ^a^	2.600	2.995	2.797 ^a^
	Mean	1.112 ^B^	1.223 ^A^	***1.167 ^A^***	2.426 ^B^	2.726 ^A^	***2.576 ^A^***
	**Mean (F)**	**0.936 ^B^**	**1.007 ^A^**		**2.038 ^B^**	**2.216 ^A^**	
ANOVA	**df**		***p*-value of the main effects and their interactions**				
Irrigation (I)	2		<0.001		<0.001		
Foliar (F)	1		<0.001		<0.001		
Hybrids (H)	5		<0.001		<0.001		
I × F	2		<0.001		<0.001		
I × H	10		<0.001		<0.001		
F × H	5		<0.001		<0.001		
I × F × H	10		<0.001		<0.001		

^†^ Cont. is the control of which distilled water provided with the equivalent amount of ethyl alcohol was sprayed for control plants instead of the EBR_24_ solution. * Means followed by different letters in the same direction differ significantly by LSD (*p* < 0.05), bold and italic means belong to the three irrigation regimes, and bold and not italic means belong to the foliar applications.

## Data Availability

The data presented in this study are available upon request from the corresponding author.

## References

[1] Kumar A., Verma J.P. (2018). Does plant-microbe interaction confer stress tolerance in plants: A review?. Microbiol. Res..

[2] Abd El-Mageed T.A., Semida W.M., Rady M.M. (2017). Moringa leaf extract as biostimulant improves water use efficiency, physio-biochemical attributes of squash plants under deficit irrigation. Agric. Water Manag..

[3] El-Mageed A.T.A., Semida W.M., Taha R.S., Rady M.M. (2018). Effect of summer-fall deficit irrigation on morpho-physiological, anatomical responses, fruit yield and water use efficiency of cucumber under salt affected soil. Sci. Hortic..

[4] Merwad A.M.A., Desoky E.M., Rady M.M. (2018). Response of water deficit-stressed *Vigna unguiculata* performances to silicon, proline or methionine foliar application. Sci. Hortic..

[5] Khademian R., Yaghoubian I. (2018). Growth of chick pea (*Cicer arietinum*) in response to salicylic acid under drought stress. J. Bio. Env. Sci..

[6] Rady M.M., Belal H.E.E., Gadallah F.M., Semida W.M. (2020). Selenium application in two methods promotes drought tolerance in *Solanum lycopersicum* plant by inducing the antioxidant defense system. Sci. Hortic..

[7] Taha R.S., Alharby H.F., Bamagoos A.A., Medani R.A., Rady M.M. (2020). Elevating tolerance of drought stress in *Ocimum basilicum* using pollen grains extract; a natural biostimulant by regulation of plant performance and antioxidant defense system. S. Afr. J. Bot..

[8] Dai A. (2013). Increasing drought under global warming in observations and models. Nat. Clim. Chang..

[9] Mansour E., Abdul-Hamid M.I., Yasin M.T., Qabil N., Attia A. (2017). Identifying drought-tolerant genotypes of barley and their responses to various irrigation levels in a Mediterranean environment. Agric. Water Manag..

[10] Mansour E., Moustafa E.A., Qabil N., Abdelsalam A., Wafa H.A., El Kenawy A., Casas A.M., Igartua E. (2018). Assessing different barley growth habits under Egyptian conditions for enhancing climate change resilience. Field Crops Res..

[11] Blum A. (2005). Drought resistance, water-use efficiency, and yield potential—Are they compatible, dissonant, or mutually exclusive?. Aust. J. Agric. Res..

[12] Lopes M.S., Araus J.L., Van Heerden P.D.R., Foyer C.H. (2011). Enhancing drought tolerance in C4 crops. J. Exp. Bot..

[13] Blum A. (2011). Plant Breeding for Water-Limited Environments.

[14] Sairam R.K., Deshmukh P.S., Saxna D.C. (1998). Role of antioxidant systems in wheat genotype tolerance to water stress. Biol. Plant..

[15] Rios J.J., Martínez-Ballesta M.C., Ruiz J.M., Blasco B., Carvajal M. (2017). Siliconmediated improvement in plant salinity tolerance: The role of aquaporins. Front. Plant Sci..

[16] Yoshida K., Terashima I., Noguchi K. (2007). Up-regulation of mitochondrial alternative oxidase concomitant with chloroplast over-reduction by excess light. Plant Cell Physiol..

[17] Huang W., Zhang S.B., Cao K.F. (2011). Cyclic electron flow plays an important role in photoprotection of tropical trees illuminated at temporal chilling temperature. Plant Cell Physiol..

[18] Ivanov A.G., Rosso D., Savitch L.V., Stachula P., Rosembert M., Oquist G., Hurry V., Hüner N.P.A. (2012). Implications of alternative electron sinks in increased resistance of PSII and PSI photochemistry to high light stress in cold-acclimated *Arabidopsis thaliana*. Photosynth. Res..

[19] Hu W.H., Yan X.H., He Y., Xi R. (2019). 24-epibrassinolide alleviate drought-induced photoinhibition in Capsicum annuum via up-regulation of AOX pathway. Sci. Hortic..

[20] Pires M.V., de Castro E.M., de Freitas B.S.M., Lira J.M.S., Magalhães P.C., Pereira M.P. (2020). Yield-related phenotypic traits of drought resistant maize genotypes. Environ. Exp. Bot..

[21] FAO (2020). Food and Agriculture Organization of the United Nations.

[22] Todaka D., Shinozaki K., Yamaguchi-Shinozaki K. (2015). Recent advances in the dissection of drought-stress regulatory networks and strategies for development of drought-tolerant transgenic rice plants. Front. Plant Sci..

[23] Rajsekhar D., Singh V.P., Mishra A.K. (2015). Integrated drought causality, hazard, and vulnerability assessment for future socioeconomic scenarios: An information theory perspective. J. Geophys. Res. Atmos..

[24] Song H., Li Y., Zhou L., Xu Z., Zhou G. (2018). Maize leaf functional responses to drought episode and rewatering. Agric. For Meteorol..

[25] Shirinbayan S., Khosravi H., Malakouti M.J. (2019). Alleviation of drought stress in maize (*Zea mays*) by inoculation with Azotobacter strains isolated from semi-arid regions. Appl. Soil Ecol..

[26] Desoky E.M., Mansour E., Yasin M.A.T., El-Sobky E.E.A., Rady M.M. (2020). Improvement of drought tolerance in five different cultivars of *Vicia faba* with foliar application of ascorbic acid or silicon. Span. J. Agric. Res..

[27] Ashraf M., Akram N.A. (2009). Improving salinity tolerance of plants through conventional breeding and genetic engineering: An analytical comparison. Biotechnol. Adv..

[28] Filgueiras L., Silva R., Almeida I., Vidal M., Baldani J.I., Meneses C.H.S.G. (2020). *Gluconacetobacter diazotrophicus* mitigates drought stress in *Oryza sativa* L.. Plant Soil..

[29] Al-Elwany O.A.A.I., Mohamed G.F., Abdurrahman H.A., Rady M.M., Abdel Latef A.A. (2020). Exogenous glutathione-mediated tolerance to deficit irrigation in salt-affected *Capsicum frutescence* (L.) plants is connected with higher antioxidant content and ionic homeostasis. Not. Bot. Horti Agrobot. Cluj-Napoca..

[30] Shakirova F., Allagulova C., Maslennikova D., Fedorova K., Yuldashev R., Lubyanova A., Bezrukova M., Avalbaev A. (2016). Involvement of dehydrins in 24-epibrassinolide-induced protection of wheat plants against drought stress. Plant Physiol. Biochem..

[31] Zhao G., Xu H., Zhang P., Su X., Zhao H. (2017). Effects of 24-epibrassinolide on photosynthesis and Rubisco activase gene expression in *Triticum aestivum* L. seedlings under a combination of drought and heat stress. Plant Growth Regul..

[32] Riboldi L.B., Múrcia J.A.G., da Cruz Araújo S.H., de Camargo e Castro P.R. (2018). The 24-epibrassinolide induces rice tolerance to water stress overcoming losses in grain yield. Aust. J. Crop Sci..

[33] Mohammadi M., Pouryousef M., Tavakoli A., Fard E.M. (2019). Improvement in photosynthesis, seed yield and protein content of common bean (*Phaseolus vulgaris*) by foliar application of 24-epibrassinolide under drought stress. Crop Pasture Sci..

[34] Mohammadi H., Akhondzadeh M., Ghorbanpour M., Aghaee A. (2020). Physiological responses and secondary metabolite ingredients in sage plants induced by 24-epibrassinolide foliar application under different water deficit regimes. Sci. Hortic..

[35] Nolan T.M., Vukasinovic’ N., Liu D., Russinova E., Yin Y. (2020). Brassinosteroids: Multidimensional regulators of plant growth, development, and stress responses—A review. Plant Cell.

[36] Tanveer M., Shahzad B., Sharma A., Khan E.A. (2019). 24-Epibrassinolide application in plants: An implication for improving drought stress tolerance in plants—A review. Plant Physiol. Biochem..

[37] Bajguz A. (2000). Effect of brassinosteroids on nucleic acids and protein content in cultured cells of *Chlorella vulgaris*. Plant Physiol. Biochem..

[38] Li Y., Liu Y., Xu X., Jin M., An L., Zhang H. (2012). Effect of 24-epibrassinolide on drought stress-induced changes in *Chorispora bungeana*. Biol. Plant.

[39] Bajguz A., Hayat S. (2009). Effects of brassinosteroids on the plant responses to environmental stresses. Plant Physiol. Biochem..

[40] Anjum S.A., Wang L.C., Farooq M., Hussain M., Xue L.L., Zou C.M. (2011). Brassinolide application improves the drought tolerance in maize through modulation of enzymatic antioxidants and leaf gas exchange. J. Agron. Crop Sci..

[41] Talaat N.B., Shawky B.T., Ibrahim A.S. (2015). Alleviation of drought induced oxidative stress in maize (*Zea mays* L.) plants by dual application of 24-epibrassinolide and spermine. Environ. Exp. Bot..

[42] Xia X.-J., Zhou Y.-H., Ding J., Shi K., Asami T., Chen Z., Yu J.-Q. (2011). Induction of systemic stress tolerance by brassinosteroid in *Cucumis sativus*. New Phytol..

[43] Attia A., El-Hendawy S., Al-Suhaibani N., Tahir M.U., Mubushar M., dos Santos Vianna M., Ullah H., Mansour E., Datta A. (2021). Sensitivity of the DSSAT model in simulating maize yield and soil carbon dynamics inarid Mediterranean climate: Effect of soil, genotype and crop management. Field Crops Res..

[44] Arnon D.I. (1949). Copper enzymes in isolated chloroplasts, polyphenoxidase in *Beta vulgaris*. Plant Physiol..

[45] Li P.M., Cai R.G., Gao H.Y., Peng T., Wang Z.L. (2007). Partitioning of excitation energy in two wheat cultivars with different grain protein contents grown under three nitrogen applications in the field. Physiol. Plant..

[46] Maxwell K., Johnson G.N. (2000). Chlorophyll fluorescence—A practical guide. J. Exp. Bot..

[47] Jagendorf A.T. (1956). Oxidation and reduction of pyridine nucleotides by purified chloroplasts. Biochem. Biophys. Acta.

[48] Avron M. (1960). Photophosphorylation by swiss-chard chloroplasts. Biochim. Biophys. Acta.

[49] Osman A.S., Rady M.M. (2014). Ameliorative effects of sulphur and humic acid on the growth, antioxidant levels, and yields of pea (*Pisum sativum* L.) plants grown in reclaimed saline soil. J. Hortic. Sci. Biotechnol..

[50] Rady M.M. (2011). Effect of 24-epibrassinolide on growth, yield, antioxidant system and cadmium content of bean (*Phaseolus vulgaris* L.) plants under salinity and cadmium stress. Sci. Hortic..

[51] Heath R.L., Packer L. (1968). Photo peroxidation isolated chloroplasts: Kinetics and stoichiometry of fatty acid peroxidation. Arch. Biochem. Biophys..

[52] Irigoyen J.J., Emerich D.W., Sanchez-Diaz M. (1992). Water stress induced changes in the concentrations of proline and total soluble sugars in nodulated alfalfa (*Medicago sativa*) plants. Plant Physiol..

[53] Bates L.S., Waldren R.P., Teare I.D. (1973). Rapid determination of free proline for water stress studies. Plant Soil.

[54] Chance B., Maehly A.C. (1955). Assay of catalases and peroxidases. Method Enzymol..

[55] Giannopolitis C.N., Ries S.K. (1977). Superoxide dismutases I. Occurrence in higher plants. Plant Physiol..

[56] Kijne J.W., Barker R., Molden D.J. (2003). Water Productivity in Agriculture: Limits and Opportunities for Improvement.

[57] Pereira L.S., Cordery I., Iacovides I. (2012). Improved indicators of water use performance and productivity for sustainable water conservation and saving. Agric. Water Manag..

[58] Fernández J.E., Alcon F., Diaz-Espejo A., Hernandez-Santana V., Cuevas M.V. (2020). Water use indicators and economic analysis for on-farm irrigation decision: A case study of a super high density olive tree orchard. Agric. Water Manag..

[59] James L.G. (1988). Principles of Farm Irrigation Systems Design.

[60] Carmer S.G., Walker W.M. (1988). Significance from the statistician’s viewpoint. J. Prod. Agric..

[61] Silva R., Filgueiras L., Santos B., Coelho M., Silva M., Estrada-Bonilla G., Vidal M., Baldani J.I., Meneses C. (2020). *Gluconacetobacter diazotrophicus* changes the molecular mechanisms of root development in *Oryza sativa* L. growing under water stress. Int. J. Mol. Sci..

[62] Ha Y., Shang Y., Nam K.H. (2016). Brassinosteroids modulate ABA-induced stomatal closure in *Arabidopsis*. J. Exp. Bot..

[63] Hernández J.A., Almansa M.S. (2002). Short-term effects of salt stress on antioxidant systems and leaf water relations of pea leaves. Physiol. Plant..

[64] Khan M., Panda S. (2008). Alterations in root lipid peroxidation and antioxidative responses in two rice hybrids under NaCl-salinity stress. Acta Physiol. Plant..

[65] Eraslan F., Inal A., Savasturk O., Gunes A. (2007). Changes in antioxidative system and membrane damage of lettuce in response to salinity and boron toxicity. Sci. Hortic..

[66] Gunes A., Cicek N., Inal A., Alpaslan M., Eraslan F., Guneri E., Guzelordu T. (2006). Genotypic response of chickpea (*Cicer arietinum* L.) hybrids to drought stress implemented at pre-and post-anthesis stages and its relations with nutrient uptake and efficiency. Plant Soil Environ..

[67] Talaat N.B., Shawky B.T. (2013). 24-Epibrassinolide alleviates salt-induced inhibition of productivity by increasing nutrients and compatible solutes accumulation and enhancing antioxidant system in wheat (*Triticum aestivum* L.). Acta Physiol. Plant..

[68] Thussagunpanit J., Jutamanee K., Sonjaroon W., Kaveeta L., Chai-Arree W., Pankean P., Suksamrarn A. (2015). Effects of brassinosteroid and brassinosteroid mimic on photosynthetic efficiency and rice yield under heat stress. Photosynthetica.

[69] Prisco J.T. (1980). Alguns aspectos da fisiologia do estresse salino. Rev. Bras. Botânica.

[70] Stępień P., Kłobus G. (2006). Water relations and photosynthesis in *Cucumis sativus* L. leaves under salt stress. Biol. Plant..

[71] Shahid M.A., Balal R.M., Pervez M.A., Abbas T., Aqeel M.A., Riaz A., Mattson N.S. (2015). Exogenous 24-Epibrassinolide elevates the salt tolerance potential of pea (*Pisum sativum* L.) by improving osmotic adjustment capacity and leaf water relations. J. Plant Nutr..

[72] Lima J.V., Lobato A.K.S. (2017). Brassinosteroids improve photosystem II efficiency, gas exchange, antioxidant enzymes and growth of cowpea plants exposed to water deficit. Physiol. Mol. Biol. Plants.

[73] Farooq M., Wahid A., Basra S.M.A. (2009). Improving water relations and gas exchange with brassinosteroids in rice under drought stress. J. Agron. Crop Sci..

[74] Sasse J.M. (2003). Physiological actions of brassinosteroids: An update. J. Plant Growth Regul..

[75] Jiang Y., Huang N. (2001). Drought and Heat stress injury to two cool season turf grasses in relation to antioxidant metabolism and lipid peroxidation. Crop Sci..

[76] Alireza Y., Aboueshaghi R.S., Dehnavi M.M., Balouchi H. (2014). Effect of micronutrients foliar application on grain qualitative characteristics and some physiological traits of bean (*Phaseolus vulgaris* L.) under drought stress. Indian J. Fundam. Appl. Life Sci..

[77] Behnamnia M., Kalantari K.M., Ziaie J. (2009). The effects of brassinosteroid on the induction of biochemical changes in *Lycopersicon esculentum* under drought stress. Turk. J. Bot..

[78] Hayashi H., Alia L.M., Deshnium P., Ida M., Murata N. (1997). Murata transformation of *Arabidopsis thaliana* with the coda gene for choline oxidasa: Accumulation of glycine betaine and enhanced tolerance to salt and cold stress. Plant J..

[79] Hebers K., Sonnewald V. (1998). Altered gene expression: Brought about by inter and pathogen interactions. J. Plant Res..

[80] Zhu J.K. (2001). Plant salt tolerance. Trends Plant Sci..

[81] Kishor P.K., Sangam S., Amrutha R.N., Laxmi P.S., Naidu K.R., Rao K.R.S.S., Rao S., Reddy K.J., Theriappan P., Sreenivasulu N. (2005). Review: Regulation of proline biosynthesis, degradation, uptake and transport in higher plants: Its implications in plant growth and abiotic stress tolerance. Curr. Sci..

[82] Rady M.M., Elrys A.S., Abo El-Maati M.F., Desoky E.M. (2019). Interplaying roles of silicon and proline effectively improve salt and cadmium stress tolerance in *Phaseolus vulgaris* plant. Plant Physiol. Biochem..

[83] Chen Z., Wang Z., Yang Y., Li M., Xu B. (2018). Abscisic acid and brassinolide combined application synergistically enhances drought tolerance and photosynthesis of tall fescue under water stress. Sci. Hortic..

[84] Mittler R. (2002). Oxidative stress, antioxidants and stress tolerance. Trends Plant Sci..

[85] Hameed M., Ashraf M., Naz N. (2011). Anatomical and physiological characteristics relating to ionic relations in some salt tolerant grasses from the Salt Range, Pakistan. Acta Physiol. Plant..

[86] Choe S. (2006). Brassinosteroid biosynthesis and inactivation. Physiol. Plant..

[87] Fang Y., Xiong L. (2015). General mechanisms of drought response and their application in drought resistance improvement in plants. Cell Mol. Life Sci..

[88] Ali M.H., Talukder M.S.U. (2008). Increasing water productivity in crop production-A synthesis. Agric. Water Manag..

[89] Hoque M.A., Banu M.N., Okuma E., Amako K., Nakamura Y., Shimoishi Y., Murata Y. (2007). Exogenous proline and glycinebetaine increase NaCl-induced ascorbate-glutathione cycle enzyme activities, and proline improves salt tolerance more than glycinebetaine in tobacco Bright Yellow-2 suspension-cultured cells. J. Plant Physiol..

[90] Şen A. (2012). Oxidative stress studies in plant tissue culture. Antioxid. Enzym..

[91] Ranieri A., Castagna A., Scebba F., Careri M., Zagnoni I., Predieri G., Pagliari M., di Toppi L.S. (2005). Oxidative stress and phytochelatin characterisation in bread wheat exposed to cadmium excess. Plant Physiol. Biochem..

[92] Kusvuran S., Kiran S., Ellialtioglu S.S., Shanker A.K. (2016). Antioxidant enzyme activities and abiotic stress tolerance relationship in vegetable crops. Abiotic and Biotic Stress in Plants—Recent Advances and Future Perspectives.

[93] Adamu C., Kumar B.N., Rajkumara S., Patil B.R., Patil H.Y., Kuligod V.B. (2014). Physiological response, molecular analysis and water use efficiency of maize (*Zea mays* L.) hybrids grown under various irrigation regimes. Afr. J. Biotechnol..

[94] Khan M.B., Yousaf F., Hussain M., Haq M.W., Lee D.J., Farooq M. (2012). Influence of planting methods on root development, crop productivity and water use efficiency in maize hybrids. Chil. J. Agric. Res..

[95] Nagore M.L., Della Maggiora A., Andrade F.H., Echarte L. (2017). Water use efficiency for grain yield in an old and two more recent maize hybrids. Field Crops Res..

[96] Hao B., Xue Q., Marek T.H., Jessup K.E., Becker J.D., Hou X., Xu W., Bynum E.D., Bean B.W., Colaizzi P.D. (2019). Grain yield, evapotranspiration, and water-use efficiency of maize hybrids differing in drought tolerance. Irrigation Sci..

[97] Allaby M. (2006). A Dictionary of Plant Sciences.

[98] Dordas C.A., Papathanasiou F., Lithourgidis A., Petrevska J.K., Papadopoulos I., Pankou C., Gekas F., Ninou E., Mylonas I., Sistanis I. (2018). Evaluation of physiological characteristics as selection criteria for drought tolerance in maize inbred lines and their hybrids. Maydica.

[99] Hussain H.A., Men S., Hussain S., Chen Y., Ali S., Zhang S., Zhang K., Li Y., Xu Q., Liao C. (2019). Interactive effects of drought and heat stresses on morpho-physiological attributes, yield, nutrient uptake and oxidative status in maize hybrids. Sci. Rep..

[100] Füzy A., Kovács R., Cseresnyés I., Parádi I., Szili-Kovács T., Kelemen B., Rajkai K., Takács T. (2019). Selection of plant physiological parameters to detect stress effects in pot experiments using principal component analysis. Acta Physiol. Plant..

[101] Mansour E., Moustafa E.S., Desoky E.-S.M., Ali M., Yasin M.A., Attia A., Alsuhaibani N., Tahir M.U., El-Hendawy S. (2020). Multidimensional evaluation for detecting salt tolerance of bread wheat genotypes under actual saline field growing conditions. Plants.

[102] Mansour E., Desoky E.M., Ali M.M.A., Abdul-Hamid M.I., Ullah H., Attia A., Datta A. (2021). Identifying drought-tolerant genotypes of faba bean and their agro-physiological responses to different water regimes in an arid Mediterranean environment. Agric. Water Manag..

